# Superconducting Quantum Interference in Twisted van der Waals Heterostructures

**DOI:** 10.1021/acs.nanolett.1c00152

**Published:** 2021-08-16

**Authors:** Liam S. Farrar, Aimee Nevill, Zhen Jieh Lim, Geetha Balakrishnan, Sara Dale, Simon J. Bending

**Affiliations:** † Department of Physics, University of Bath, Bath BA2 7AY, United Kingdom; ‡ Department of Physics, University of Warwick, Coventry CV4 7AL, United Kingdom

**Keywords:** Van der Waal heterostructures, Josephson junction, Superconducting quantum interference device, Two-dimensional materials, NbSe_2_

## Abstract

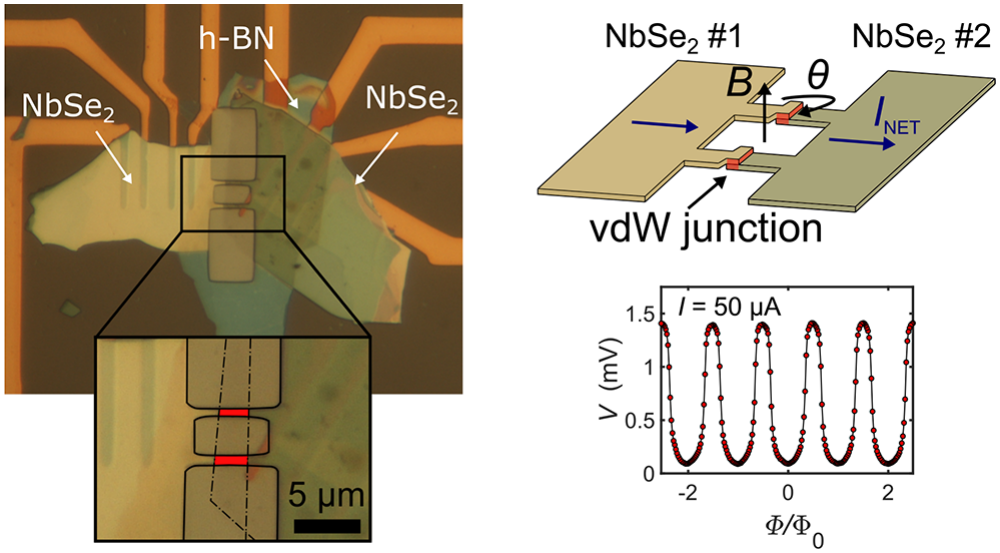

We demonstrate the formation of both Josephson junctions and superconducting quantum interference devices (SQUIDs) using a dry transfer technique to stack and deterministically misalign mechanically exfoliated flakes of NbSe_2_. The current–voltage characteristics of the resulting twisted NbSe_2_–NbSe_2_ junctions are found to be sensitive to the misalignment angle of the crystallographic axes, opening up a new control parameter for optimization of the device performance, which is not available in thin-film-deposited junctions. A single lithographic process has then been implemented to shape Josephson junctions into SQUID geometries with typical loop areas of ∼25 μm^2^ and weak links ∼600 nm wide. At *T* = 3.75 K in an applied magnetic field, these devices display large stable current and voltage modulation depths of up to Δ*I*_c_ ∼ 75% and Δ*V* ∼ 1.4 mV, respectively.

Superconducting quantum interference devices (SQUIDs) are key components in the development of ultrasensitive electric and magnetic measurement systems.^1^ The basic SQUID design consists of a superconducting ring intersected by one (rf SQUID) or two (dc SQUID) Josephson junctions; the latter consists of two superconducting electrodes coupled by weak links that allow the flow of supercurrent.^2,3^ The weak link can take a variety of forms including point contacts, physical constrictions, or heterostructures consisting of a thin normal metal or insulating barrier separating the two superconductors.^4^ The latter type is typically fabricated by deposition of metallic superconductors such as Al and Nb, with the tunneling barrier formed by an oxide layer.^5,6^ This oxide barrier varies in thickness on the atomic scale and often contains defect traps, which can lead to highly nonuniform supercurrent distributions.^7^ In addition, over time, oxygen atoms in the oxide barrier can diffuse out, altering the normal state resistance of the junction resulting in a variation of the critical current, a process that can be detrimental to the longevity of the device.^8^ Since Josephson junctions are the basis of many superconducting technologies such as qubits,^9^ quantum metrology,^10^ and superconducting quantum interference devices (SQUIDs), the development and incorporation of new materials with improved properties and functionality are vital.

An alternative route to the fabrication of oxide-free Josephson junctions is the stacking of two-dimensional materials (2D) into vertical van der Waals (vdW) heterostructures,^11^ a technique which holds promise for creating atomically clean and defect free interfaces. Additionally, vdW-based devices provide access to a wide variety of crystalline superconducting materials such as Bi_2_Sr_2_CaCu_2_O_8+δ_,^12^ FeSe,^13^ and 2*H*-NbSe_2_.^14^ These materials all share a common characteristic in that they have a layered structure and easily cleave perpendicular to the *c*-axis. These properties, along with the availability of dry transfer techniques,^15^ have allowed the fabrication of vdW heterostructure devices comprised of two or more mechanically exfoliated flakes. Such devices include junctions with dielectric tunneling barriers,^16,17^ superconducting-normal-superconducting Josephson junctions,^18^ and van der Waals interface Josephson junctions,^19^ which may prove useful in the formation of superconducting qubits.^20^ Furthermore, a vdW-based device offers an additional variable in the form of controlling the relative twist angle between the crystallographic axes of the two materials;^21^ a degree of freedom which is not available in conventional heterostructures. This twisting creates a misalignment between the two crystals, changing the atomic registry at the interface and leading to angular-dependent interlayer interactions. This enables one to tune the electronic coupling via the twist angle, leading to effects such as band hybridization,^22,23^ minigaps and band replicas due to scattering on moiré potentials,^24^ charge transfer and changes in effective masses,^25^ and has led to the discovery of a moiré superlattice in graphene/h-BN,^26^ as well as electrically tunable superconductivity in twisted bilayer graphene.^27^ This new field of study, named twistronics, may prove valuable in the incorporation of two-dimensional materials into more complex vdW superconducting devices such as qubits and SQUIDs.

In this study, twisted NbSe_2_–NbSe_2_ heterostructures are formed by stacking two NbSe_2_ flakes with a well-determined misalignment of the crystallographic axes using the procedure described in Methods and highlighted in Figure 1a. Briefly, two NbSe_2_ flakes are sequentially stacked with a small overlap onto a Si/SiO_2_ substrate with prepatterned Au contacts and encapsulated with a thin layer of hexagonal boron nitride. Typical junction areas are on the order of 20–60 μm^2^ (Figure 1b,c).

**Figure 1:**
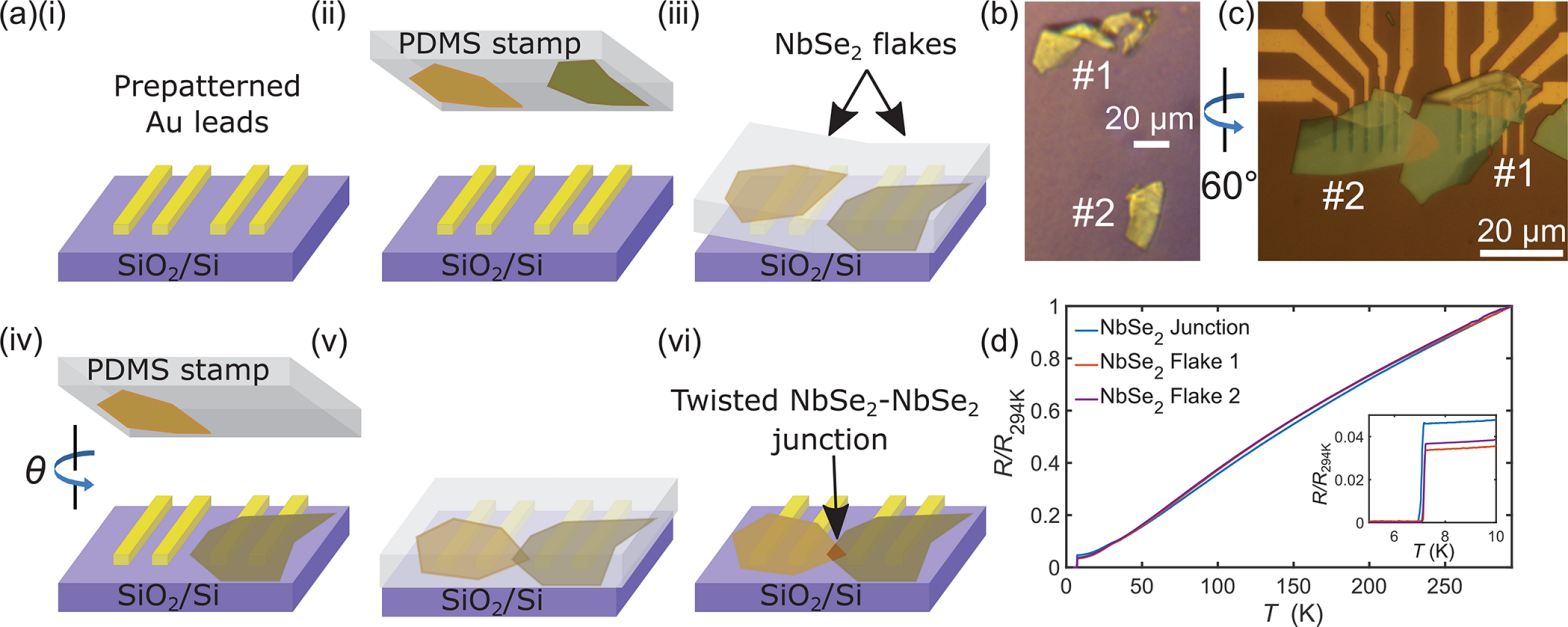
(a) Schematic of the device fabrication method. (i) Au contacts are deposited onto a Si/SiO_2_ substrate. (ii) A single exfoliation is made of a bulk NbSe_2_ crystal, and the resulting flakes are transferred onto a PDMS stamp. (iii) The PDMS is brought into contact with the substrate, which is tilted at a small angle. The PDMS is slowly pressed into the substrate until the boundary of the PDMS-substrate contact region lies beyond one of the flakes. (iv) The PDMS is then retracted, leaving the first flake deposited onto the Au contacts. The substrate is then rotated such that the crystallographic axes of the two NbSe_2_ flakes are now misaligned by an angle θ. (v) The second flake is positioned above the first, and the PDMS is brought into contact with the substrate. (vi) The PDMS is now retracted, leaving the second flake contacting both the first flake and the Au contacts. (b) Optical micrograph of two NbSe_2_ flakes on a PDMS stamp. (c) Optical micrograph of the two overlapping NbSe_2_ flakes on SiO_2_/Si. Here one of the flakes (labeled *#*2) has been rotated by 60°. (d) Temperature-dependence of the normalized resistance *R*(*T*)/*R*(294 K) for the two NbSe_2_ flakes and the overlapping junction region between them. The inset shows an expanded view of the superconducting transitions.

Before describing the characterization of devices patterned with SQUID geometries, we investigate the transport properties of twisted NbSe_2_–NbSe_2_ junctions. The temperature-dependence of the normalized four-point resistance *R*(*T*)/*R*(294 K) from 4 to 294 K is shown in Figure 1d for two NbSe_2_ flakes with a relative twist angle of 60°, as well as the resulting junction formed by the overlap region. We note that there is an uncertainty of ±1° in the misalignment angle of devices arising from both the resolution of the rotation stage and unwanted movement of the PDMS during the stamping progress. Both flakes and junction show metallic transport behavior, which is phonon-limited at high temperature (*R* ∝ *T*) and disorder limited at low temperature (*R* approaches a constant value) before reaching the superconducting state at *T*_c_ ∼ 7 K.^28^ As the *T*_c_ of NbSe_2_ is known to reduce in flakes <10 layers,^16,17^ all flakes used were at least 10 nm thick, ensuring negligible suppression of *T*_c_. The residual resistance ratio (RRR), defined as *R*(*T* = 294 K)/*R*(*T* = 8 K), is found to be ∼30 for the two NbSe_2_ flakes, with a small variation due to the slightly different flake thicknesses. This is in contrast to the RRR of the junction region which is found to vary between ∼10–25 depending on the twist angle (see Figure S1 in the Supporting Information). A close up of the superconducting transition is shown in Figure 1d, where it can be seen that the junction displays a slightly broader transition width when compared to the individual NbSe_2_ flakes, indicative of additional disorder at the interface.

## Josephson Junction Performance

**Figure 2:**
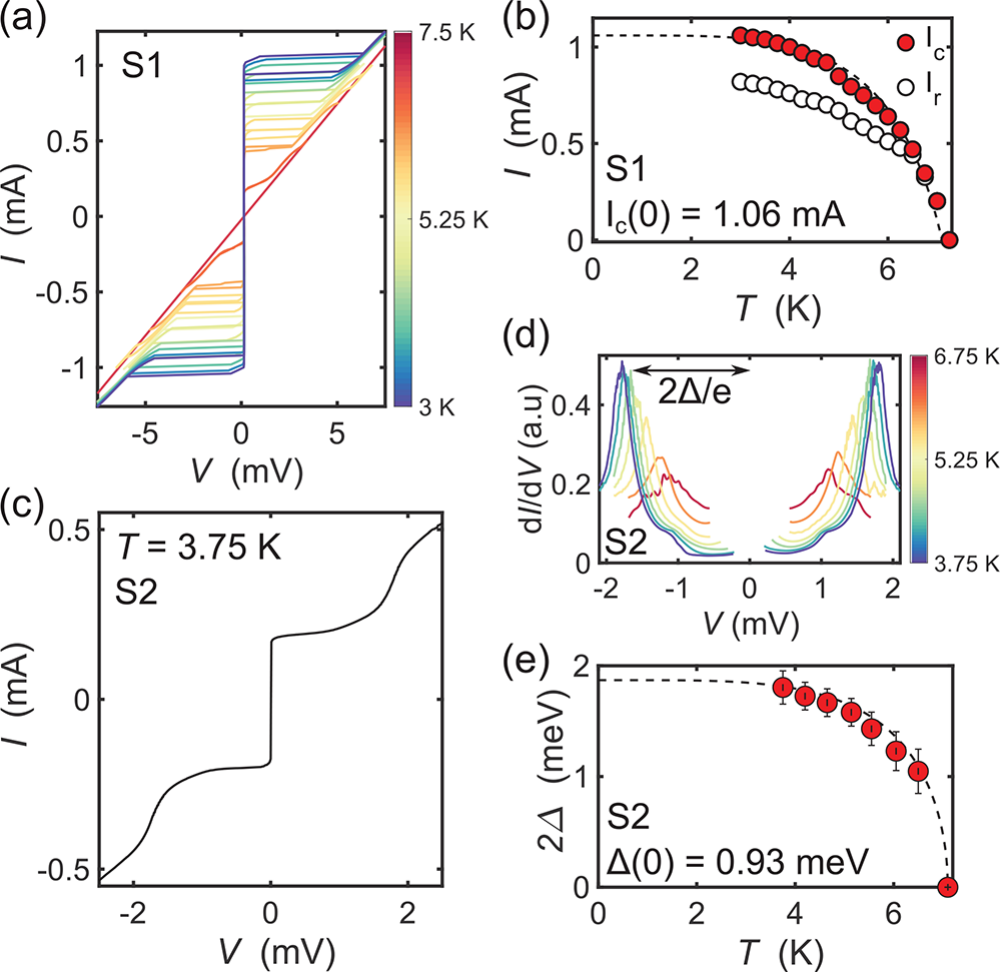
(a) Current–voltage (*I*–*V*) characteristics of a NbSe_2_–NbSe_2_ junction (sample S1, θ ≈ 6°) for temperatures between *T* = 3 and 7.5 K. (b) Temperature dependence of the critical current *I*_c_ (red) and retrapping current (white). The dashed line is calculated from AB theory (see text). (c) *I*–*V* characteristics for sample S2 (θ ≈ 20°) at *T* = 3.75 K. (d) Temperature dependence of d*I*/d*V* versus V between *T* = 3.75 and 6.75 K. (e) Temperature dependence of the estimated superconducting gap Δ. The dashed line is calculated from an interpolation approximation to the BCS theory.

Next, we examine the current–voltage (*I*–*V*) characteristics of a NbSe_2_–NbSe_2_ junction for temperatures *T* = 3 to 7.5 K (S1, Figure 2a). Here the misorientation angle of the junction is estimated to be θ ∼ 6° from comparison of optical micrographs of the two flakes. From the presence of distinct retrapping currents in the *I*–*V* curves, it is clear that the junction behavior is typical of an underdamped Josephson junction. This indicates that the vdW interface decouples the superconducting order parameters and creates a weak link that allows the flow of a Josephson supercurrent. In agreement with the conclusions of Yabuki et al.^19^ we also find that our devices are reasonably well described by the RCSJ model indicating that they are operating in the superconducting tunnel junction limit. Each NbSe_2_ flake has multiple electrical contacts, allowing the use of different voltage lead configurations to confirm that the bulk critical current of each flake is substantially larger than that of the junction. Assuming that the vdW barrier is insulating, the temperature dependence of the critical current, *I*_c_, can be calculated using the Ambegaokar–Baratoff (AB) theory,^29^ in which *I*_c_(*T*) is expressed as
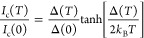
1where *I*_c_(0) and Δ(0) are the zero-temperature critical current and superconducting gap, respectively. Δ(*T*) can be described by an interpolation approximation to the Bardeen–Cooper–Schrieffer (BCS) theory temperature-dependent superconducting energy gap:
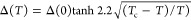
2

The fit in Figure 2b shows that the critical current of sample S1 is reasonably well described by AB theory, in agreement with previous reports on NbSe_2_ Josephson junctions,^19^ despite the assumption of a symmetric single gap superconductor. This allows us to estimate Δ(0) = 0.90 mV and *I*_c_(0) = 1.06 mA.

However, not all devices fabricated behave as underdamped junctions, and upon examination of a second device presented in Figure 2c (S2, θ ∼ 20°), we observe *I*–*V* characteristics which are non hysteretic, indicative of a junction which is in the strongly overdamped regime (β_c_ < 1). Here, nonlinear features arise due to quasiparticle currents that reveal information about the superconducting density of states. We examine this in Figure 2d by taking the numerical differential of the measured *I*–*V* curves. From this, we observe an intricate non-BCS-like gap structure, with peaks located at ±1.79 mV at *T* = 3.75 K. Furthermore, we observe a shoulder-like hump at lower energy (±1 mV), similar to that seen in tunneling spectroscopy of NbSe_2_ using van der Waals tunnel barriers.^16,17^ Using the BCS approximation in eq 2, we can extract the zero-temperature superconducting gap Δ(0) = 0.93 meV as shown in Figure 2e.

## Angular Dependence

**Figure 3:**
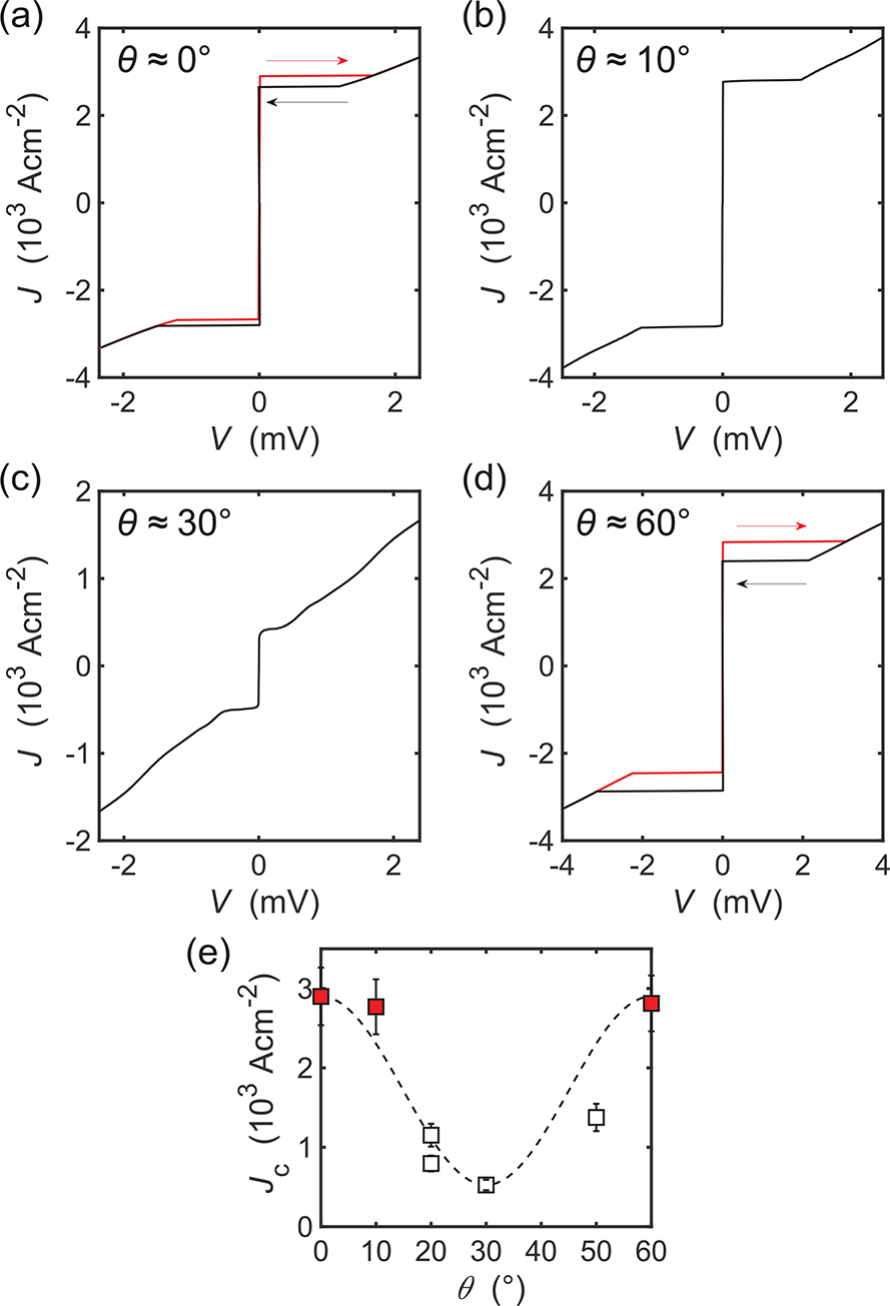
(a) Current density–voltage (*J*–*V*) characteristics for twisted NbSe_2_–NbSe_2_ junctions with twist angles θ ≈ 0°, (b) 10°, (c) 30°, (d) 60°. The red and black arrows indicate the direction of the current sweep. (e) Variation of the critical current density of 7 NbSe_2_–NbSe_2_ junctions with twist angles between 0°–60°. Devices that displayed hysteretic (nonhysteretic) *J*–*V* characteristics are represented by closed (open) symbols. The dashed line is a guide to the eye. All measurements were recorded at *T* = 3.75 K.

Having observed strikingly different characteristics in NbSe_2_–NbSe_2_ junctions prepared using the same fabrication method, we turn our attention to the role of twist angle in these devices. Using the method presented in Figure 1a, we are able to fabricate devices with a determined misalignment angle relative to the crystallographic axes of the two flakes. The results are shown in Figure 3a–d, in which we present the current density–voltage (*J*–*V*) characteristics of four NbSe_2_–NbSe_2_ junctions with twist angles in the range θ ≈ 0–60°. First, we examine the θ ≈ 0° device, in which hysteresis is observed in the *J*–*V* characteristics, indicating the junction is an underdamped Josephson junction. In samples with larger twist angles (>10°, Figure 3b), this hysteresis disappears, giving rise to reversible junctions which show features of quasiparticle gap structure at angles >20° (Figure 3c). As the twist angle is increased beyond about 40°, the gap features disappear, and the hysteresis eventually returns near θ ≈ 60° (Figure 3d). In Figure 3e, we plot the critical current density *J*_c_ as a function of twist angle θ for 7 NbSe_2_ junctions. From this, it can be seen that *J*_c_ is maximum at values of θ ≈ 0° and 60°, with a minimum at 30°.

Angle-resolved photoemission spectroscopy (ARPES) data^30^ and density functional theory calculations^31^ show that there are five electronic bands crossing the Fermi energy in NbSe_2_. Of these, one is a small Se-4*p p*_*z*_-derived “pancake″-shaped hole pocket, while the other four are Nb-4*d*-derived bands with roughly cylindrical Fermi surfaces centered at the Γ and *K* points in the Brillouin zone. Based on ARPES measurements, the Se pancake has been shown to exhibit no superconducting gap, while the Nb-derived sheets display superconductivity, which is strongly anisotropic in *k*.^32,33^ This anisotropy of the superconducting order parameter is characterized by maxima in the gap at 60° intervals around the Fermi sheets. From this, we hypothesize that at twist angles close to 30°, tunneling is dominated by processes that couple regions of the Fermi surface with a maximum gap in one layer with regions of a minimum gap in the other layer. This angular-dependent selectivity in the tunneling process suppresses the critical current *I*_c_, leading to a lower value of β_c_ and nonhysteretic *I*–*V* characteristics.

The observed twist dependence could also be related to the specific atomic arrangement at the interface, which depends on the flake terminations as well as any effects arising from relative lateral displacements of the two flakes. However, the latter is expected to be negligible in our large overlap regions, which are many hundreds of unit cells wide. We note that we have minimized any variation in the interface quality and homogeneity by performing all fabrication in the inert environment provided by a nitrogen glovebox.

## SQUID Performance

**Figure 4:**
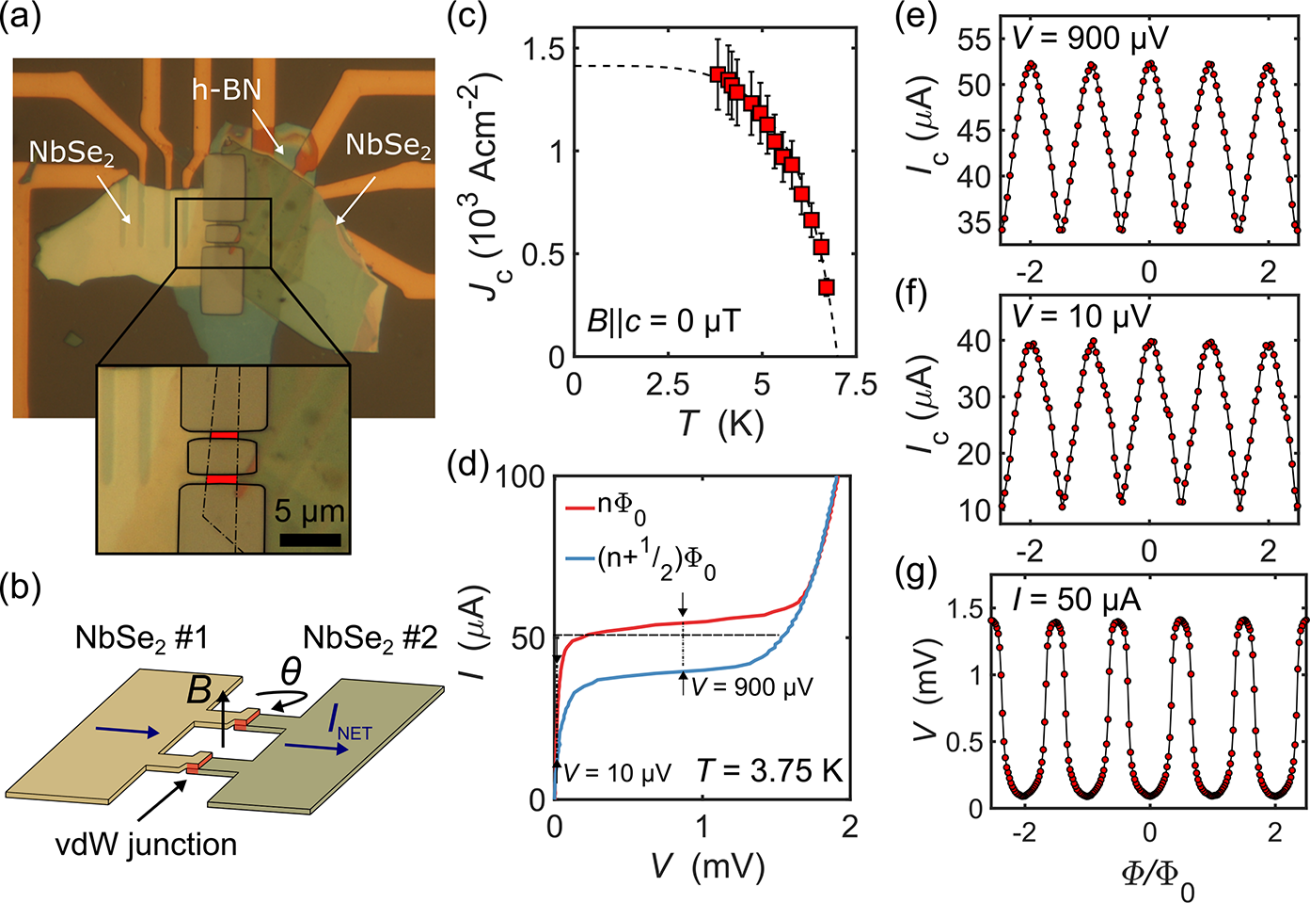
(a) Optical image of an etched SQUID structure (θ ≈ 25°). The inset shows a close up of the structure, with the overlap region between the two NbSe_2_ flakes highlighted in red. (b) Schematic diagram of the SQUID device shown in panel a. (c) Critical current as a function of temperature in zero applied field of the SQUID device shown in panel a. The dashed line is calculated from the AB theory (see text). (d) Current–voltage (*I*–*V*) characteristics at *T* = 3.75 K of the device shown in panel a. The red (blue) curve corresponds to the maximum (minimum) value of the positive critical current *I*_c_ within one period. (e) Modulation of *I*_c_ as a function of the applied magnetic flux under a voltage bias of *V* = 900 μV (f) and *V* = 10 μV at *T* = 3.75 K. (g) Voltage modulation as a function of the magnetic flux under a current bias of *I* = 50 μA at *T* = 3.75 K. A horizontal shift has been applied to panels (e-g) to account for the Earth’s magnetic field.

Having established the ability to fabricate high quality nonhysteretic Josephson junctions, we examine the ability to use them in more complex superconducting devices such as superconducting quantum interference devices (SQUIDs). Junction devices were patterned into a SQUID geometry using reactive ion etching as described in Methods. An image of a typical device is shown in Figure 4a with a schematic shown in Figure 4b and consists of a superconducting loop between two ∼600 nm wide Josephson junctions formed in the overlap region of the two NbSe_2_ flakes (2–3 μm). The SQUID loop is of width, *W* ≈ 3.5 μm, and length, *L* ≈ 7.0 μm, creating an internal hole of area ≈24.5 μm^2^.

When an external magnetic field *B* ∥_c_ is applied, phase shifts are induced between the two junctions leading to interference and a net critical current that oscillates with a period *B*_0_ = Φ_0_/*A*_eff_, where *A*_eff_ includes corrections to the geometrical area of the superconducting loop due to effects such as flux focusing. To characterize the effect of an external magnetic field on our NbSe_2_–NbSe_2_ SQUIDs, we have performed electrical transport measurements at temperatures down to *T* = 3.75 K. Figure 4c shows the temperature dependence of the critical current calculated using AB theory. The extracted zero-temperature critical current density is found to be *J*_c_(0) = 1.41 Acm^–2^ using Δ(0) = 0.96 meV. We observed no reduction of the critical temperature, indicating that etching caused no measurable deterioration of the junction quality. Figure 4d shows the current–voltage characteristics for a NbSe_2_ SQUID, with the two curves corresponding to the maximum, , and minimum, , measured values of the critical current within a single oscillation period. The modulation of the critical current, *I*_c_(Φ) for two different voltage set points, is shown in Figure 4e,f, where Φ is the applied magnetic flux. Under a bias voltage of *V* = 900 μV, the current modulation depth is 33%, which increases to 75% for a bias of *V* = 10 μV. The noise level of the signal increased slightly at *V* = 10 μV due to small temperature oscillations arising from the refrigeration cycle of the closed-cycle cryostat (see Supporting Information for further voltage/current set points, a discussion of device asymmetry, and results from a second SQUID). The inductance parameter characterizes the amplitude of the current modulation and is given by β_L_ = 2*LI*_c_/Φ_0_,^34^ where Φ_0_ is the superconducting flux quantum and *L* is the inductance of the SQUID loop. Fitting our measured Δ*I*_c_ data to a numerical model,^35^ we estimate that β_L_ ≈ 2 and *L* ≈ 30 pH, which is a factor ∼2 large than other estimates of the self-inductance based on the SQUID geometry.^35^ The area of the SQUID loop is 24.5 μm^2^, leading to a theoretical value of *B*_0_ = ≃84 μT, assuming no corrections due to flux focusing, etc. This theoretical value is close to the measured value of *B*_0_ = 78 μT.

An important property of SQUIDs is the realization of nonhysteretic *I*–*V* characteristics (β_c_ < 1), allowing the use of flux-locked loop feedback schemes to minimize the flux noise.^36,37^ We are able to tune this desirable property in our NbSe_2_ SQUIDS by deterministically controlling the twist angle during device fabrication, with a wide range of angles, over which β_c_ is found to be sufficiently small. Figure 4g shows the voltage under a bias current of 50 μA, as a function of the magnetic field applied to the SQUID, revealing large voltage modulations with a depth of ≈1.4 mV. The voltage modulation depth is characterized by Δ*V* ≃ *δR*Δ*I*_c_, where Δ*I*_c_ is the critical current modulation depth, and δR is the differential resistance of the SQUID calculated here to be δR ≃ 28 Ω at the maximum value of Δ*V*, i.e., the peak of the d*V*/d*I* curve at positive voltages. A large value of Δ*V* is required to achieve low flux noise SQUID devices as it minimizes the noise contribution from the amplifier  to the white flux noise, given by , in flux-locked operation.^2,38^ No degradation in *T*_c_ or *I*_c_ of these SQUIDs was observed after multiple cooling cycles or when remeasured after storage in a nitrogen glovebox for 4 weeks, indicating that they are both stable and suitable for repeated long-term use.

## Discussion

In conclusion, we have developed a method to fabricate Josephson junctions and SQUIDs based on a twisted van der Waal heterostructure architecture. The formation of Josephson junctions is achieved using a dry transfer method and requires no wet lithographic steps. The result is a junction device whose *I*–*V* characteristics can be deterministically tuned via the twist angle. A single lithographic process is then implemented to shape the Josephson junction into a SQUID geometry with typical loop areas of ≃25 μm^2^ and weak links ≃600 nm wide. We obtain voltage modulation depths of Δ*V* ≃ 1.4 mV and current modulation depths of Δ*I* ≃ 75% from devices that display long-term stability.

Our method demonstrates the ability to fully integrate 2D materials into the design of a SQUID, paving the way for designer superconducting circuits through the incorporation of other 2D materials, each with distinct electronic properties. The utilization of van der Waals bonded circuits may also have other advantages: here, the devices are only anchored to the substrate by the weak vdW force, allowing the possible pick-up and transfer of characterized SQUIDs onto other substrates or materials, while the intrinsically thin nature of 2D materials may allow their incorporation into flexible electronic circuits.^39^ Other applications for the technology is in the design of superconducting qubits such as those based on flux qubit architectures.^40,41^ It is well-known that the performance of these is often limited by dissipation, decoherence, and noise from two-level defect systems. The single crystalline structure of 2D flakes, along with low defect densities, may provide circuit components with superior performance.

## Method

Thin flakes of NbSe_2_ were mechanically exfoliated from high-quality single crystals onto silicone elastomer polydimethylsiloxane (PDMS) stamps. Flakes of suitable geometry and thickness (∼20–50 nm) were then sequentially transferred onto Si/SiO_2_ (300 nm oxide) substrates with prepatterned Au contacts. To ensure crystallographic alignment of the twisted vdW junctions, one single exfoliation of the bulk single crystal is performed, which results in multiple thin flakes with aligned crystallographic axes. From this, two neighboring flakes were chosen with a rotation of the substrate performed after stamping the first flake. The resulting device is thus a twisted vdW junction with the two flakes misaligned by a chosen angle with ±1° accuracy. The entire dry transfer setup is housed in a nitrogen glovebox with an oxygen and moisture content <1 ppm, ensuring an oxide free interface. Devices selected for fabrication into SQUIDs were subsequently capped with a thin layer (∼20 nm) of hexagonal boron nitride (h-BN) using the same dry transfer technique. Following this, a poly(methyl methacrylate) e-beam resist was spin coated onto the substrate, which was then kept under a vacuum (10^–6^ mbar) overnight to remove any solvent. Next, standard e-beam lithography (EBL) was used to define the area of the junction to be etched to form the SQUID loop, before transfer into an inductively coupled plasma etcher. The sample was then reactively ion etched using an O_2_ + SF_6_ mixture, after which the sample was immersed in acetone to remove leftover resist before storage in a nitrogen glovebox. A total of 16 NbSe_2_ twisted junctions were measured, of which 7 were fabricated with a determined twist angle. A total of 9 SQUIDs were fabricated, 4 of which were fully characterized and showed the expected oscillatory dependence of critical current on applied magnetic field. A bias current of 1 μA was used for all resistance measurements.

## Supporting Information Available

Table of Josephson junction parameters; table of SQUID parameters; twist angle dependence of the residual resistance ratio for the NbSe_2_ devices; capacitance analysis for a typical NbSe_2_ Josephson junction; further magnetic field-dependent voltage (critical current) oscillations at varying current (voltage) set points of the SQUID; asymmetry analysis of the device; analysis of a second SQUID with larger junction area (PDF)
