# Supplementary material for: Superconducting Quantum Interference in Twisted van der Waals Heterostructures
Source: Nano Lett. 2021 Aug 16;21(16):6725–31. doi: 10.1021/acs.nanolett.1c00152 (PMC8397396; doi:10.1021/acs.nanolett.1c00152)
Supplement: Supplementary file 1 — nl1c00152_si_001.pdf [file nl1c00152_si_001.pdf]

Supporting Information: Superconducting Quantum Interference in Twisted van der Waals Heterostructures

Liam S. Farrar,[*,†] Aimee Nevill,[†] Zhen Jieh Lim,[†] Geetha Balakrishnan,[‡] Sara Dale,[†] and Simon J. Bending[†]

[†]*Department of Physics, University of Bath, Bath BA2 7AY, United Kingdom*
[‡]*Department of Physics, University of Warwick, Coventry CV4 7AL, United Kingdom*

E-mail: L.S.Farrar@bath.ac.uk

Table 1: Summary of the Josephson junction geometrical and transport parameters at T = 3.75 K.

θ (°)	I_c (mA)	R_N (Ω	A (μm^2)	J_c (10^3Acm^{-2})	V_c = $I_c R_N$ (mV)	RRR	Retrapping
$\approx$ 0	1.74	1.0	60	2.9	1.74	22.5	Y
$\approx$ 10	0.97	1.36	35	2.77	1.31	22	Y
$\approx$ 20	0.27	3.76	23	1.17	1.02	8.6	N
$\approx$ 20	0.34	3.2	43	0.79	1.09	12.4	N
$\approx$ 30	0.16	4.22	30	0.53	0.68	8.4	N
$\approx$ 50	0.62	0.9	45	1.38	0.56	12.3	N
$\approx$ 60	1.35	2.32	48	2.81	3.13	16.5	Y

Table 2: Summary of the SQUID geometrical and transport parameters at T = 3.75 K.

Name	$A_{Junction}$ (μm^2)	A_{Loop} (μm^2)	$I_c^{B=0}$ (μA)	B_0 (μT)
SQUID 1	$\approx$ 2	24.5	50	78
SQUID 2	$\approx$ 9	15.0	185	130
SQUID 3	$\approx$ 7	25.0	220	72
SQUID 4	$\approx$ 5	20.0	115	-

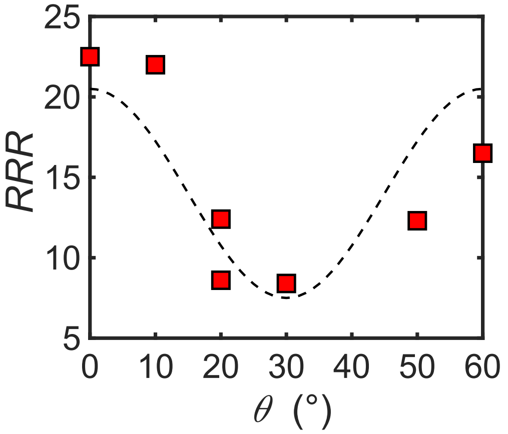

Figure S1: Residual resistance ratio (*RRR*) as a function of twist angle of the $NbSe_2$-$NbSe_2$ junctions presented in Figure 3e of the main text. Here, *RRR* is defined as the ratio between R(294K)/R(10K). The dashed line is a guide to the eye.

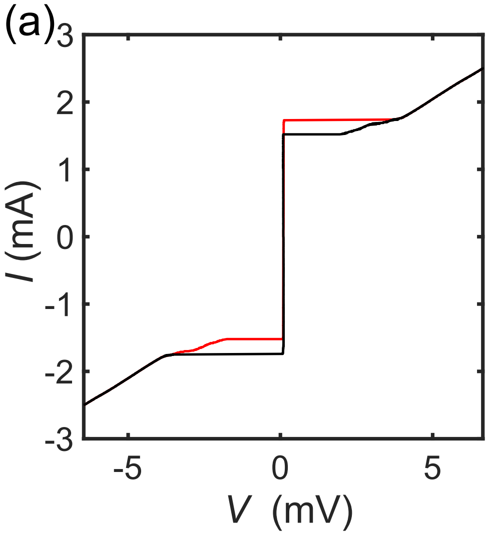

Figure S2: Current-voltage ($I-V$) characteristics at T = 3.75 K for an $NbSe_2$-$NbSe_2$ Josephson junction. To quantify the junction dynamics, we examine the McCumber parameter β_c, found by fitting the ratio between the retrapping current I_r and the critical current I_c and comparing it to a microscopic model.[S1] The critical current and critical retrapping current of the presented device is determined to be I_c = 1.74 mA and I_r = 1.52 mA respectively at T = 3.75 K, leading to a $I_\mathrm{r}/I_\mathrm{c}$ ratio of $\sim$ 0.87 and a McCumber parameter $\beta_c \approx$ 1. This can be related to the capacitance of the junction, C, using the expression $\beta_c = 2eI_cR_N^2C\hbar^{-1}$, where R_N is the normal state junction resistance, determined here to be 2.2 Ω. This leads to a junction capacitance of $C \approx$ 3.9x10^{-14} F. Assuming the permeability is that of the vaccum and using the junction area $A \approx$ 14 μm^2, the junction thickness corresponds to $\sim$ 3 nm (about 2 unit cells). In otherwise identical devices, this has previously been shown to represent a parallel plate capacitor with a vacuum gap comparable in size to the c-axis layer spacing of 2H-NbSe2.[S2]

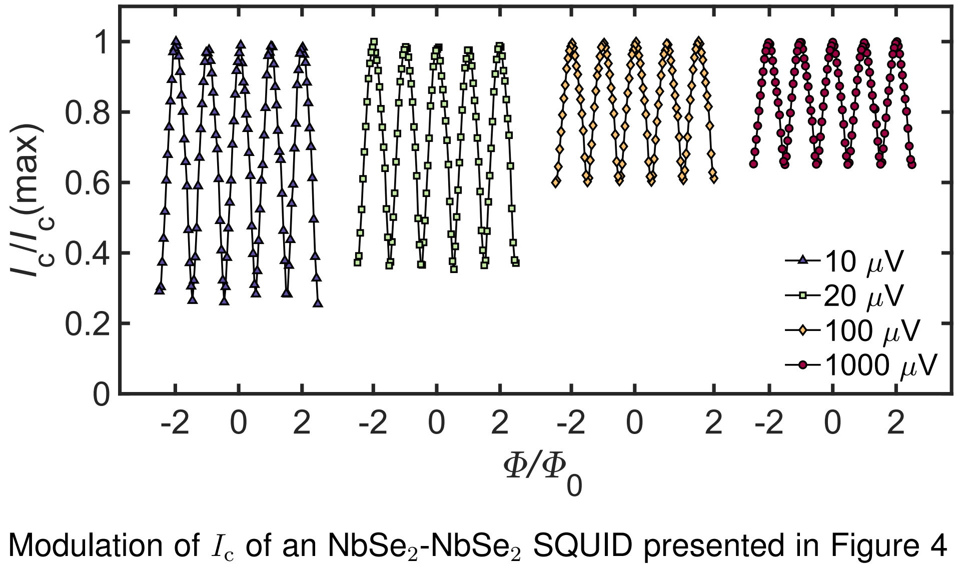

Figure S3: Modulation of I_c of an $NbSe_2$-$NbSe_2$ SQUID presented in Figure 4 of the main text, at T = 3.75 K as a function of the applied magnetic flux under various bias voltages.

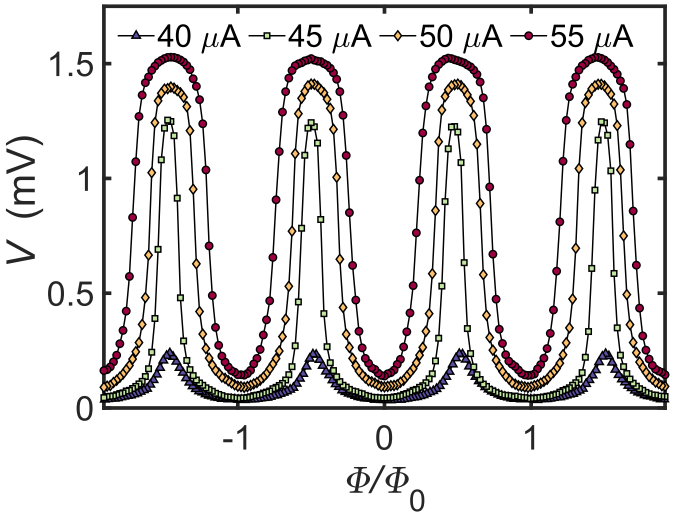

Figure S4: Voltage modulation of an $NbSe_2$-$NbSe_2$ SQUID presented in Figure 4 of the main text, at T = 3.75 K as a function of the magnetic flux under various bias currents.

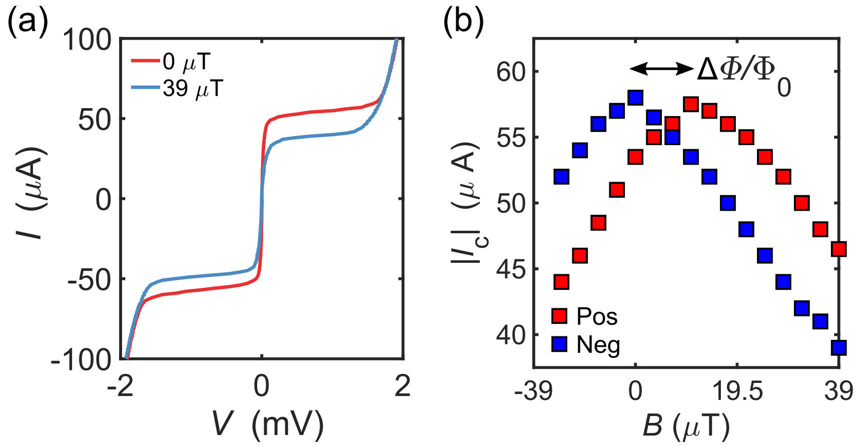

Figure S5: a) Current-voltage (I-V) characteristics at T = 3.75 K of the SQUID device shown in Figure 4. The red (blue) curve corresponds to the maximum (minimum) value of the positive critical current I_c within one period. b) Flux dependence of the modulus of the critical current of $|I_c|$ for both positive and critical critical currents. I_c is determined by the maximum in dV/dI. The maximum of the positive and negative critical current oscillations are observed to be shifted by an amount $\Delta\Phi/\Phi_0$, likely due to asymmetry in the size of the two junctions. This magnetic flux asymmetry can be described by the relation $\Delta\Phi/\Phi_0 = \alpha_I\beta_L$,[S3] where α_I is the critical current asymmetry parameter give by $I_{0,1} = I_0(1-\alpha_I)$, $I_{0,2} = I_0(1+\alpha_I)$, and β_L is the inductance parameter estimated to be $\beta_L \approx 2$. $\Delta\Phi/\Phi_0 \approx$ 0.11 leading to $\alpha_I \approx$ 5.5%.

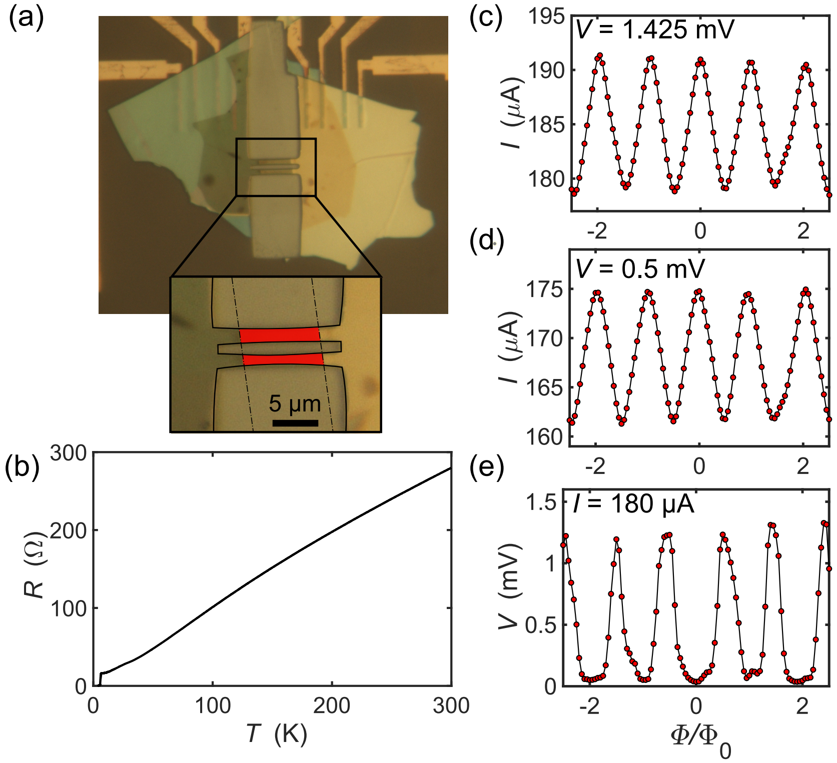

Figure S6: a) Optical image of an etched SQUID structure (θ $\approx$15°) with very large junction areas. The insert shows a close up of the structure, with the overlap region between the two $NbSe_2$ flakes highlighted in red. b) Temperature-dependence of resistance measured across the junctions of the device in a). c) Modulation of I_c as a function of the applied magnetic flux under a voltage bias of V = 1.425 V d) and V = 0.5 mV at T = 3.75 K. e) Voltage modulation as a function of the magnetic flux under a current bias of I = 180 μA at T = 3.75 K. A horizontal shift has been applied to c)-e) to account for the Earth's magnetic field.

References

[S1] Martínez-Pérez, M.J.; Koelle NanoSQUIDs: Basics & recent advances. *Phys. Sci. Rev.* **2017**, 2(8), 1-27.

[S2] Yabuki, N.; Moriya, R.; Arai, M.; Sata, Y.; Morikawa, S.; Masubuchi, S.; Machida, T. Supercurrent in van der Waals Josephson junction. *Nat. Commun.* **2016**, 7 (1), 1-5.

[S3] Muller, J.; Weiss, S.; Gross, R.; Kleiner, R.; Koelle, D. Voltage-flux-characteristics of asymmetric DC SQUIDs. *IEEE Trans. Appl. Supercond.*, **2001**, 11, 912-915
